# Supplementary material for: Drug repurposing for aging research using model organisms
Source: Aging Cell. 2017 Jun 16;16(5):1006–15. doi: 10.1111/acel.12626 (PMC5595691; doi:10.1111/acel.12626)
Supplement: Supplementary file 7 — Data S1 Zip‐Archive of all report cards. [file ACEL-16-1006-s007.zip › RC_ADN.pdf]

ADN

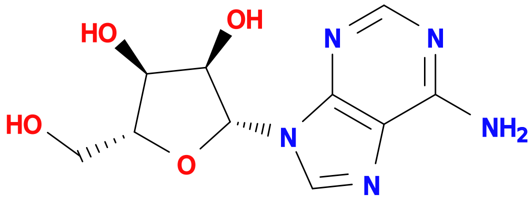

Database identifiers

ChEMBLCompound	CHEMBL477
DrugBank	DB00640
CHEBI	16335
ZINC	ZINC02169830
eMolecules	474947

Ranking

	Rank	Score
Drosophila	486/697	0.291
C. elegans	NA	NA

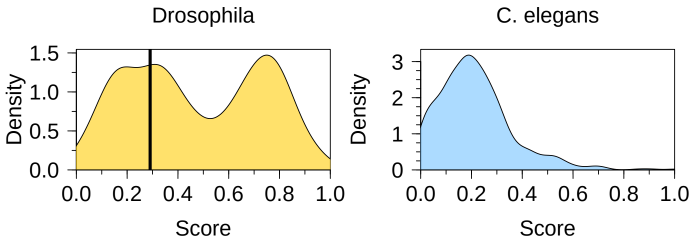

	Ageing implication	Domain conservation	Binding site conservation	Binding affinity	Bioavailability	Lipinski	Promiscuity	Purchasability	Drug approval	**Total**
Drosophila	0.203	0.909	0.978	0.562	(0.9)	0.0	-0.0	0.1	0.1	0.291
C. elegans	NA	NA	NA	NA	NA	NA	NA	NA	NA	NA

Names

- **adenosine**
- 6-Amino-9-beta-D-ribofuranosyl-9H-purine
- 9-beta-D-Ribofuranosidoadenine
- 9-beta-D-Ribofuranosyl-9H-purin-6-amine
- Ade-Rib
- Adenine Deoxyribonucleoside
- Adenocard
- Adenyldeoxyriboside
- Ado
- beta-D-Adenosine
- Deoxyadenosine

Roles

anti-arrhythmia drug, vasodilator agent, analgesic, human metabolite, fundamental metabolite, Adenosine receptor agonist

Status

Approved drug (according to ChEMBL)	Yes
Administration Route	Intravenous
Number of Rule of 5 violations	0
Binding affinity to original target in log units (RF-Score prediction)	5.25
Burns *C. elegans* bioavailability prediction	-8.84

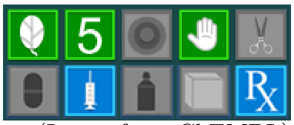

(Image from ChEMBL)

Medical Information

Indicatation: Used as an initial treatment for the termination of paroxysmal supraventricular tachycardia (PVST), including that associated with accessory bypass tracts, and is a drug of choice for terminating stable, narrow-complex supraventricular tachycardias (SVT). Also used as an adjunct to thallous chloride TI 201 myocardial perfusion scintigraphy (thallium stress test) in patients who are unable to exercise adequately, as well as an adjunct to vagal maneuvers and clinical assessment to establish a specific diagnosis of undefined, stable, narrow-complex SVT.
Mechanism of action: Adenosine slows conduction time through the AV node and can interrupt the reentry pathways through the AV node, resulting in the restoration of normal sinus rhythm in patients with paroxysmal supraventricular tachycardia (PSVT), including PSVT associated with Wolff-Parkinson-White Syndrome. This effect may be mediated through the drug's activation of cell-surface A1 and A2 adenosine receptors. Adenosine also inhibits the slow inward calcium current and activation of adenylate cyclase in smooth muscle cells, thereby causing relaxation of vascular smooth muscle. By increasing blood flow in normal coronary arteries with little or no increase in stenotic arteries (with little to no increase in stenotic arteries), adenosine produces a relative difference in thallous (thallium) chloride TI 201 uptake in myocardium supplied by normal verus stenotic coronary arteries.
Metabolism: Intracellular adenosine is rapidly metabolized either via phosphorylation to adenosine monophosphate by adenosine kinase, or via deamination to inosine by adenosine deaminase in the cytosol.
Retail: APP Pharmaceuticals, A-S Medication Solutions LLC, Astellas Pharma Inc., Baxter International Inc., Bedford Labs, Ben Venue Laboratories Inc. and 10 more suppliers
Adenosine 6 mg/2 ml vial - 8.1 USD / ml
Adenosine 12 mg/4 ml vial - 8.44 USD / ml
Adenoscan 3 mg/ml vial - 10.09 USD / ml
Adenosine powder - 15.86 USD / g
Adenosine-5 monophosphate - 19.74 USD / g

(Information from DrugBank)

Compound Target Characteristics

Proto-oncogene tyrosine-protein kinase receptor Ret

Best gene implication in ageing for this target family came from gene P07949 via mapping the annotation from Ensembl ENSG00000165731 via mapping the annotation from EntrezGene 5979 via mapping the annotation from GenAgeHuman 0056 annotated in GenAge release 17.

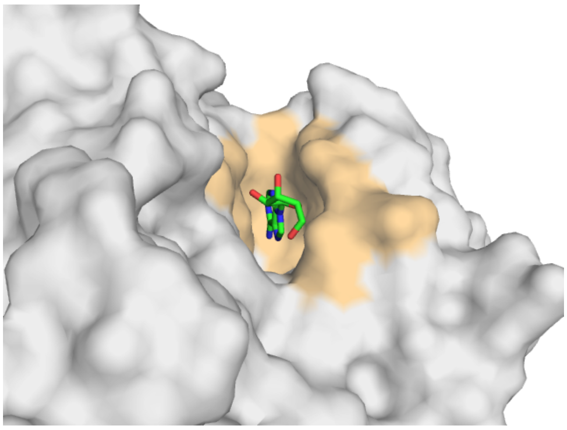

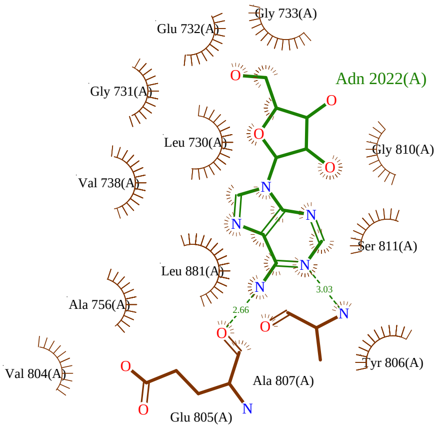

protein	amino acids contacts (binding site)
PDB:4cki:chainA:P07949	L G E G V A V E Y A G S L
PDB:4ckj:chainA:P07949	L G E G V A V E Y A G S L
tr:B4DGX8:B4DGX8_HUMAN	L G E G V A V E Y A G S L
sp:P07949:RET_HUMAN	L G E G V A V E Y A G S L
sp:G3V9H8:RET_RAT	L G E G V A V E Y A G S L
sp:P35546:RET_MOUSE	L G E G V A V E Y A G S L
tr:Q7JQD5:Q7JQD5_DROME	L G E G V A I E Y A G S L
tr:Q7KA88:Q7KA88_DROME	L G E G V A I E Y A G S L
tr:Q7KT06:Q7KT06_DROME	L G E G V A I E Y A G S L
tr:Q9BHA9:Q9BHA9_DROME	L G E G V A I E Y A G S L

	whole protein		domain-based		contact-based	
protein	ident	simil	ident	simil	ident	simil
PDB:4cki:chainA:P07949	0.99	1.0	1.0	1.0	1.0	1.0
PDB:4ckj:chainA:P07949	0.99	1.0	1.0	1.0	1.0	1.0
tr:B4DGX8:B4DGX8_HUMAN	0.73	0.73	1.0	1.0	1.0	1.0
sp:P07949:RET_HUMAN	1.0	1.0	1.0	1.0	1.0	1.0
sp:G3V9H8:RET_RAT	0.85	0.95	0.96	0.99	1.0	1.0
sp:P35546:RET_MOUSE	0.83	0.95	0.95	0.99	1.0	1.0
tr:Q7JQD5:Q7JQD5_DROME	0.17	0.39	0.55	0.83	0.92	0.98
tr:Q7KA88:Q7KA88_DROME	0.2	0.6	0.55	0.83	0.92	0.98
tr:Q7KT06:Q7KT06_DROME	0.2	0.6	0.55	0.83	0.92	0.98
tr:Q9BHA9:Q9BHA9_DROME	0.2	0.6	0.55	0.83	0.92	0.98

Ret (FBgn0011829) associated phenotypes

decreased cell death, heat sensitive, lethal - all die before end of pupal stage, partially lethal - majority die, size defective, somatic clone

(Information from FlyBase)
